# How should we prioritise global surgery? A capabilities approach argument for the place of surgery within every health system

**DOI:** 10.1136/bmjgh-2023-013100

**Published:** 2023-11-10

**Authors:** Rashi Jhunjhunwala, Sridhar Venkatapuram

**Affiliations:** 1 BIDMC Dept of Surgery, Harvard Medical School, Boston, Massachusetts, USA; 2 King's Global Health Institute, King's College London, London, UK

**Keywords:** Surgery, Health policy, Health systems

## Abstract

In the rapidly evolving landscape of global health issues and policy, surgery has historically been sidelined due to concerns about high cost, complexity and other concerns including quantitatively less surgical disease burden in comparison to infectious disease or other health conditions. Now, in the context of pandemics, climate change, shrinking health budgets and other global health security concerns, the hard-won progress in raising the profile of surgical care is at risk, and a reconceptualisation is needed to maintain its position in global healthcare agendas. We challenge the long-standing ethical frameworks that underlie healthcare priority setting, namely cost-effectiveness analysis and human rights, that have contributed to surgery being sidelined for decades. They incompletely account for improvements to life quality and well-being that are possible through surgical healthcare systems. We argue for the Capabilities Approach as an alternative normative framework because it emphasises the moral importance of supporting every person’s abilities to be and to do the things they value. Through this framework, we can produce a more comprehensive conception of healthcare that goes beyond biomedical health, and surgical healthcare would ultimately gain a higher priority in valuation of healthcare and non-healthcare interventions.

**Summary box**

- The Capabilities Approach is an ethical framework that focuses on every person’s ability to live lives that they value, and it can be used to consider questions of priority setting in healthcare, and can also provide a more comprehensive conception of healthcare that includes more than biomedical health outcomes.
- Surgical healthcare has historically been neglected in the global health policy space due to misconceptions about the high cost and complexity of surgical interventions.
- Over the last decade, ethical frameworks that are often used in healthcare priority setting, such as cost-effectiveness analysis and human rights, have been used to justify investment into surgical healthcare with some effect, but more robust justification is needed.
- Through this model, surgical healthcare would ultimately gain a higher priority position in the valuation of healthcare and non-healthcare interventions and policies because it centres on enabling people to live lives that they value.

## Introduction

Surgery has historically been sidelined in evolving global health agendas for reasons such as cost, complexity of surgical illnesses, higher visibility diseases, or rapidly scalable vertical disease control programmes.^1^ Against this challenging background, efforts have been made to identify cost-effective surgical procedures and craft national level surgical policy plans. Moreover, a proposal just recently adopted at the 76th World Health Assembly (WHA) in May 2023 repositions surgical healthcare as a component of emergency, critical and operative care (ECO).^2^ Since the current global health policy space is contentious and crowded with issues like climate change, pandemics, conflicts, shrinking health budgets and political instability, repositioning surgery within ECO might be strategically advantageous. However, positioning surgery primarily as emergency care also severely curtails surgery’s scope and impact.

Surgery is crucially important in emergencies as well as critical in many other ways that support healthcare’s moral purpose to help us pursue good quality lives. Aside from responding to emergency threats to life, and controlling disease, even the WHO now recognises that a third pillar (i.e., purpose) of our health policy efforts must be geared toward enabling people to enjoy better health and well-being—and all people, not just better-off people in rich countries.^3^ A better understanding is needed of the place of surgical healthcare within health systems. Addressing the backlog of non-emergency surgical procedures due to COVID-19 lockdowns worldwide requires a paradigm shift in understanding and approach to surgical healthcare. A new kind of thinking about surgery is also an opportunity to re-evaluate the ends and means of the broader healthcare system, and particularly the scarcity mindset prevalent in global health policymaking.^4^

## Global surgery in the 21st century

Before 2000, few global health researchers or advocates considered surgery relevant to discourses on effective, efficient, and equitable health systems. Policy arguments for surgical healthcare as a component of strong health systems and as a component of universal health coverage (UHC) were made in earnest only beginning in 2006.^5–8^ In 2015 the WHA adopted Resolution WHA68.15 recognising the value of strengthening infrastructure for emergency and essential surgical and anaesthesia care as well as for equity centred access and delivery.^9^ The same year, the Lancet Commission on Global Surgery produced a report asserting that over 5.3 billion people worldwide were unable to access needed surgical care, particularly in low- and middle-income countries (LMICs).^10^ Also in 2015, the third edition of the Disease Control Priorities (DCP3) included a standalone volume identifying 43 essential surgical procedures for all health systems.^11^ Surgical healthcare has also been argued to contribute to various Sustainable Development Goals (SDGs) as well as in realising the ethical principle of leaving no one behind.^12^ These various efforts, and research further dispelling misconceptions about need, complexity and high cost of surgical interventions^7 8^ have been catalysing a global movement for surgical system strengthening.

Nevertheless, multiple factors still uniquely hamper development of surgical healthcare infrastructure in LMICs on top of the post-COVID-19 challenges facing all health systems, the UHC agenda, and SDGs.^13^ For example, while many nations have developed national surgical policies, securing adequate funding–either domestic or foreign–for implementation continues to be difficult.^14^ An even more fundamental challenge is the operationalising of largely conceptual National Surgical Anaesthesia and Obstetric Plans into the local capability to deliver surgical interventions such as the 43 identified by DCP3. Concrete surgical service delivery entails increasing training for care providers, developing equipment and medication supply chains and improving infrastructure for reliable delivery of electricity and water, among other actions. As such, surgical healthcare policies must be integrated into national priorities for healthcare initiatives, infrastructure and technological innovation.^15^

Without question, health technology assessment, cost-effectiveness analysis (CEA) and health metrics like quality-adjusted life years (QALYs) and disability-adjusted life years (DALYs) have greatly improved healthcare policymaking. Yet, the underlying utilitarian ethical reasoning that aims only for maximising certain valued outcomes has consistently marginalised surgery. Indeed, ethical frameworks are not often explicitly identified or discussed in priority-setting exercises. Ethical values, alongside assumptions about human rationality, often go unquestioned while being presented as ‘technical’ or ‘rational’ principles, especially when discussing the health needs of poor people. Ethics underlie all decisions made about who can access which types of care, when, where and with how much out-of-pocket expenditure.

We are adding our voices to support the use of the Capabilities Approach (CA) to design, implement, and evaluate policies targeting quality of life, health and specifically here, surgical access. The CA’s central idea is that a good human life is not reducible to one thing (eg, happiness, wealth, liberty); a good human life consists of the abilities to be and do many different things, largely facilitated through social conditions over each person’s life course.^16^ Healthcare interventions (HCIs) are one part of diverse social conditions that help individuals become capable of planning and pursuing the kinds of lives they want and value. And, importantly, the CA asserts that every single person has a moral claim to the basic capabilities of living a good life. This framework does not immediately pit specific HCIs against one another, reduce health into a single metric, or assert that one particular distribution pattern of benefits is always right. Rather, it provides an analytical framework for reasoning about the value of different components of human health, and guides inclusive deliberations and priority-setting in healthcare. The CA framework also enables policymakers to recognise the broad capacities of surgical healthcare to provide life-affirming care and address multiple dimensions of health justice that are currently excluded in decision-making.

## Why have other approaches to priority-setting not promoted surgical healthcare?

CEA and human rights (HR) are two ethical frameworks that often drive investment in global health policy. CEA follows the utilitarian ethic of producing ‘the greatest good for the greatest number’ while an HR approach to health asserts certain state obligations towards citizens. CEA in surgery was first applied to a surgical platform in Bangladesh, showing the cost-effectiveness of surgical interventions and jumpstarting the movement to contest prevalent ideas of surgery as an expensive luxury.^17^

However, CEA methods cannot adequately account for the complexity and nuance of surgical disease. Many surgical conditions contributing to high rates of morbidity and mortality, especially in LMICs, are relatively straightforward–cases of trauma, postpartum haemorrhage or acute abdominal emergency. Other complex conditions may also be addressed by surgery but at different stages in the disease process or at varying levels of severity. The difficulty in establishing a definition of ‘surgical disease’ for which surgery is the solution is one of the very reasons why it has been challenging to put surgery on the priority-setting map. It also highlights why focusing largely on the ‘emergency’ aspect of surgical disease is limiting. Many diseases and other conditions may be treated non-surgically but require the availability of surgical providers for when the case requires emergent surgical intervention. For example, the management of septic shock or a complicated compromised airway requires surgical backup being available.^18^ The decision regarding which HCIs to include in a ‘basic’ or ‘essential’ package based on CEA may exclude patients with complex presentations that could be addressed by a simple surgical procedure.

CEA is a conceptually accessible methodology for conducting priority-setting. It provides straightforward guidance by identifying and prioritising the interventions that produce greatest numbers of disease-free and disability-free years of life across populations for the least cost. This can be especially helpful when balancing competing claims in settings of limited national resources. However, aggregation of health benefits across people tends to disadvantage individuals who are severely disabled, older people, those with multiple morbidities and others. Maximisation of health units also excludes conceptions of the moral purpose of health that are not based in utility.^19^ With regard to surgery, CEA frameworks can exclude procedures that are socially valued but do not easily figure in a utilitarian calculus. For example, complex and life-sustaining congenital heart surgery is often socially supported but is irreconcilable with CEA alone. These procedures are often done using justification based in HR. In fact, HR has often been the only ethical language to counter the utilitarian maximisation prevalent in healthcare priority-setting.

The language and framework of HR in relation to health gained traction as the global community began to discuss global gender inequalities in health,^20^ and broadened the scope of what is valued in health policy. HR allowed for practical definitions of health to expand beyond the biomedical model, and conceptualised health promotion based in international law which carried actionable weight.^21^ HR theory also provides compelling language for health advocacy. ‘Rights’ are central to Western ideas of liberty and freedom, and capitalising on this language pulls health into a morally relatable sphere. However, HR approaches to health can be stymied in national and social contexts in which community claims are prioritised over individual claims.

As with many aspects of global health, well-intentioned efforts to address global surgical needs are largely being led by Global North institutions and individuals who endorse a liberal theory of individual rights. Voices of LMIC patients and other actors are largely absent in the places where many of these healthcare prioritisation platforms are intended to be implemented, though this is changing.^22^ Pursuing the HR framework with its individual rights focus while dismissing non-individualistic social values is no longer feasible or advisable.^23^ Priority-setting using a framework based on European enlightenment-defined human dignity and moral claims is unlikely to resonate with those who hold other substantive moral views. Without engaging with those whom these policies apply in the policymaking process, it is impossible to know whether HR are relevant and compelling. Without inclusive deliberations, there is less potential for legitimate, relevant and ethically justifiable decisions to be made.^24^

## The capability approach and surgical healthcare

Following efforts in other public policy domains, we propose the CA as an alternative ethical framework for priority-setting in healthcare. The CA’s emphasis on diversity of human life and values creates a paradigm in which a range of possibilities for ‘a good life’ and good health can be supported rather than one singular conception. When the capability to be healthy (CH) is seen as a meta-capability that enables other basic capabilities for a decent human life,^25^ this concept can act as a foundation on which to consider questions of priority in policymaking. Rather than deliberating only on whether to fund specific surgical interventions, a CA-based prioritisation framework would emphasise the importance of access to surgical evaluation as a component of the capability to be healthy—and not, as it is now positioned, as an intervention in competition with other HCIs. Considering the framework of capabilities does not deny the value of CEA as additional priority-setting criteria. Using CA provides a foundation for both CEA criteria and respect for individual dignity (rights) to be used in conjunction with each other, more so than either alone.

We provide two examples of CA-derived healthcare priority-setting criteria: ‘economic productivity’ and ‘ability to care for others.’ A CH-based system of priority-setting incorporates related non-biological benefits because to be healthy includes the capability to have control over one’s immediate physical environment. Such a capability often requires the liberty and opportunity to work and earn income as a means of securing other freedoms. Furthermore, supporting economic productivity of individuals helps maintain a stable economy and positively impacts the collective social and environmental components of society. This is not to say that ultimate priority should be given to interventions that promote economic productivity; this would be unfair to individuals who cannot be economically productive and contribute this indirect health benefit to society.^26^

Thus, we propose the criterion of ‘ability to care for others’. In many societies a large sector of the population, often girls and women, provide crucial care to other community members. This caregiver relationship often results in detriments to the health of the female caregiver,^27^ many of whom report feeling the burden of providing care more keenly than their male counterparts.^28^ Nevertheless, the ability to care for others, help maintain family and community relationships, and the security of knowing that individuals will be cared for by their community can also be thought of as vital components of living a valuable life. Yet, our societies could do better to mitigate caregiving’s heavy burden and harmful impacts.

To put these two capability criteria in context of surgery, traumatic injuries that require a laparotomy, orthopaedic stabilisation or amputation tend to affect the socially worst-off and often result in catastrophic financial hardship. Treating these injuries through surgical care would be elevated in priority by the ability to restore a patient’s economic productivity as well as the ability to care for others. Likewise, consider reproductive health. Unplanned or unwanted pregnancies can hinder a person’s ability to work or care for family and community members. Elective termination of pregnancy and elective surgical sterilisation would protect and expand freedoms in planning and pursuing family and other life plans.

## Healthcare as a means to living a life of value

The CA, focused on a decent human life for every person, is a coherent, universal model from which many types of priority-setting criteria can be specified. This approach can augment other prevalent approaches striving to integrate stakeholder perspectives in determining quality of procedural outcomes.^29^ The CA provides a universally recognisable theoretical basis from which to specify relevant criteria based on the variety of human experience and pertinent human capabilities. The priority level of various HCIs would change considering these expanded criteria. In reality, priority setting in healthcare often uses multiple ethical and philosophical frameworks. It is easy to imagine that after evaluation using CEA, the additional criteria of economic productivity and caring for others would push many surgical interventions into higher-priority ranking. And, the fundamental focus on human dignity that unites the CA and HR can emphasise the importance of seeing each individual as and end unto themselves; each with agency and the right to live a life they value.

The CA has helpful implications for surgical and other types of healthcare and offers benefits to other components of societal development. The emphasis on innovation and infrastructure as well as concern for economic growth in the SDGs aligns with the inevitable expansion of roads, sanitation systems, and jobs that would accompany the increased prioritisation of surgical healthcare. Investing in a surgical healthcare delivery platform will also contribute to economic growth by facilitating the development and maintenance of a stable workforce.^30^ In fact, given the higher burden of surgically-treatable injuries and childbirth complications on those who are most likely to constitute the workforce, it may be impossible for certain nations to improve their economic standing without this type of healthcare investment.^31^ In order to operationalise the CA and understand the extent of the impact a CA can have in health, metrics are being developed and assessed that aim to identify the best way to use capability instruments in health economic evaluations.^32^ As these instruments are still in the development phase, there is no current consensus on how best to describe improvements in health and human functioning using capabilities-based metrics. However, this type of investigation is crucial in working towards a way in which to expand our understanding of how to incorporate CA into a more holistic assessment of the intersection of healthcare with other sectors that impact people’s lives. Importantly, the CA and metrics derived from it can serve to further facilitate ‘legitimate’ decisions about specific procedures that are relevant to their populations, constructed from coherent, moral and rigorous theoretical bases that focus on the lives people want to live, conceived beyond biomedical or surgical outcomes.

## Conclusion

We have made three claims in this analysis. First, as a global health community, we must reimagine surgery within a context that does not reduce healthcare to realising a set of narrow, emergency or individual biological outcomes. The existing systems of healthcare priority setting do not sufficiently account for broader non-biological outcomes that people worldwide value. It is also misguided to perpetuate the view that surgery is a highly resource-intensive endeavour that is a luxury for rich people in rich countries. Second, the CA as a method helps rethink the role of surgery within health systems and will prioritise surgery higher as well as recenter on healthcare’s moral purpose of helping people plan and pursue good quality lives. Third, more implicitly, we have argued that it is no longer acceptable that any socially marginalised population should be subjected to minimalist or ‘just enough’ health interventions, particularly by distant policy experts.

## Data Availability

There are no data in this work.
